# Author Correction: Sequence specificity analysis of the SETD2 protein lysine methyltransferase and discovery of a SETD2 super-substrate

**DOI:** 10.1038/s42003-020-01351-z

**Published:** 2020-10-19

**Authors:** Maren Kirstin Schuhmacher, Serap Beldar, Mina S. Khella, Alexander Bröhm, Jan Ludwig, Wolfram Tempel, Sara Weirich, Jinrong Min, Albert Jeltsch

**Affiliations:** 1 Institute of Biochemistry and Technical Biochemistry, University of Stuttgart, Allmandring 31, 70569 Stuttgart, Germany; 2 Structural Genomics Consortium, University of Toronto, 101 College Street, Toronto, ON M5G 1L7 Canada; 3 Biochemistry Department, Faculty of Pharmacy, Ain Shams University, African Union Organization Street, Abbassia, Cairo, 11566 Egypt

**Keywords:** Transferases, Methylases, Enzyme mechanisms, Methylation, X-ray crystallography

Correction to: *Communications Biology* 10.1038/s42003-020-01223-6, published online 16 September 2020.

In the originally published version of the Article, equations 2 and 3 were incorrectly given as follows [equation 2: $v = \frac{{v_{{\mathrm{max}}}}}{{c_{\mathrm{s}} + K_{\mathrm{M}}}}$; equation 3: $v = \frac{{v_{{\mathrm{max}}}}}{{c_{\mathrm{s}} + \,K_{\mathrm{M}}( {{\mathrm{1}} \,+ \frac{{c_{\mathrm{I}}}}{{K_{\mathrm{I}}}}})}}$]. The correct equations are [equation 2: $v = \frac{{v_{{\mathrm{max}}}{\mathrm{c}}_{\mathrm{s}}}}{{c_{\mathrm{s}} + \,K_{\mathrm{M}}}}$; equation 3: $v = \frac{{v_{{\mathrm{max}}}{\mathrm{c}}_{\mathrm{s}}}}{{c_{\mathrm{s}} + \,K_{\mathrm{M}}( {{\mathrm{1}} + \,\frac{{c_{\mathrm{I}}}}{{K_{\mathrm{I}}}}} )}}$]. All analyses were conducted with the correct equations. The errors have been corrected in the HTML and PDF versions of the Article.

