# Supplementary material for: Sequence specificity analysis of the SETD2 protein lysine methyltransferase and discovery of a SETD2 super-substrate
Source: Commun Biol. 2020 Sep 16;3:511. doi: 10.1038/s42003-020-01223-6 (PMC7495481; doi:10.1038/s42003-020-01223-6)
Supplement: Supplementary file 1 — Supplementary Information [file 42003_2020_1223_MOESM1_ESM.pdf]

Sequence specificity analysis of the SETD2 protein lysine methyltransferase and discovery of a SETD2 super-substrate

Maren Kirstin Schuhmacher, Serap Beldar, Mina S. Khella, Alexander Bröhm, Jan Ludwig, Wolfram Tempel, Sara Weirich, Jinrong Min & Albert Jeltsch

Supplementary Figures

Supplementary Fig. 1: SETD2 protein and catalytic activity.

Supplementary Fig. 2: Error analysis of the peptide array results shown in Fig. 1.

Supplementary Fig. 3: Additional kinetic analyses with the ssK36 and H3K36 peptides

Supplementary Fig. 4: Quantification of the data shown in Fig. 3a.

Supplementary Fig. 5: Quantification of the data shown in Fig. 3b.

Supplementary Fig. 6: Investigation of the methylation of putative non-histone protein substrates of SETD2.

Supplementary Fig. 7: Simulated annealing omit map with the whole peptide omitted.

Supplementary Fig. 8: Hydrogen bonds and hydrophobic interactions likely to be formed between the SETD2 catalytic domain and the bound peptides.

Supplementary Fig. 9: Control experiments related to Fig. 6c.

Supplementary tables

Supplementary Table 1: Information related to the peptide array shown in Fig. 2a.

Supplementary Table 2: Information related to the peptide array shown in Fig. 3a.

Supplementary Table 3: Information related to the peptide array shown in Fig. 3b.

Supplementary Figures

Supplementary Fig. 1: SETD2 protein and catalytic activity.

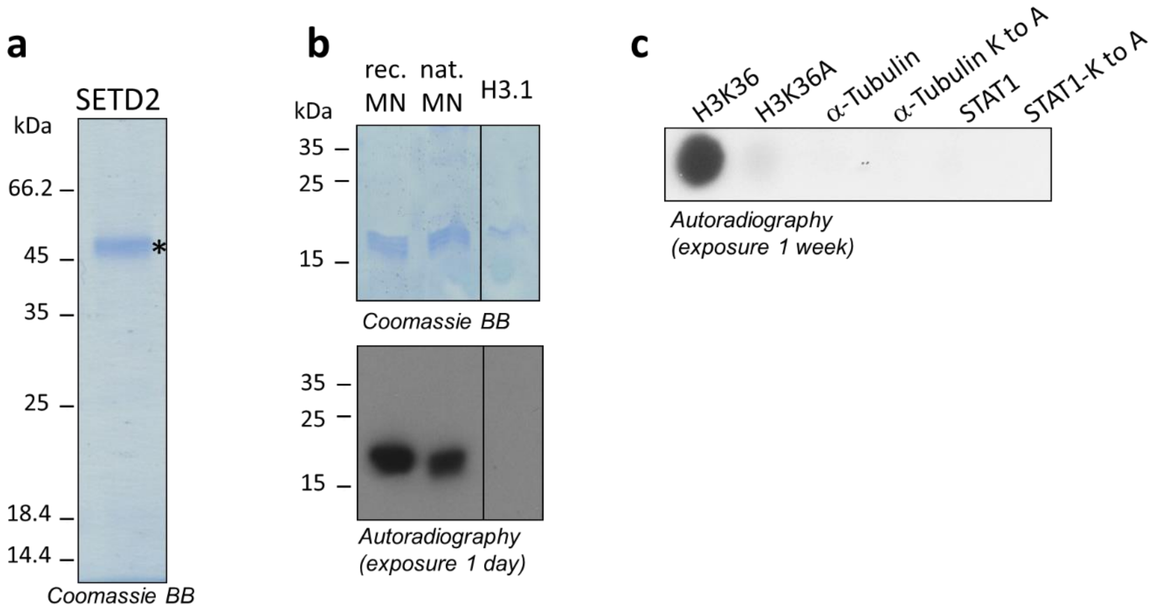

a Purified His_6-tagged SETD2 SET domain (1347-1711) was separated on an SDS-gel and stained with Coomassie BB. The asterisk indicates the band of SETD2 (50 kDa). **b** Methylation of reconstituted mononucleosomes (rec. MN), native mononucleosomes isolated from HEK393 cells (nat. MN) and H3.1 protein (NEB) by SETD2. The substrates were incubated in methylation buffer containing SETD2 and radioactively labeled AdoMet, separated on an SDS-gel and the methylation signal was captured by autoradiography. The upper panel shows the Coomassie stained gel as loading control, the lower panel the autoradiography image. Both parts of the images were taken from the same original gel and they are shown at equal brightness and contrast. **c)** Methylation of peptide SPOT arrays by SETD2. H3K36 (29-43), α-tubulin (33-47), STAT1 (518-532) and their corresponding target lysine to alanine mutant peptides were synthesized on a cellulose membrane. The membrane was incubated in methylation buffer supplemented with radioactively labeled AdoMet and SETD2. The signal of the transferred radioactive methyl groups was visualized by autoradiography.

Supplementary Fig. 2: Error analysis of the peptide array results shown in Fig. 1.

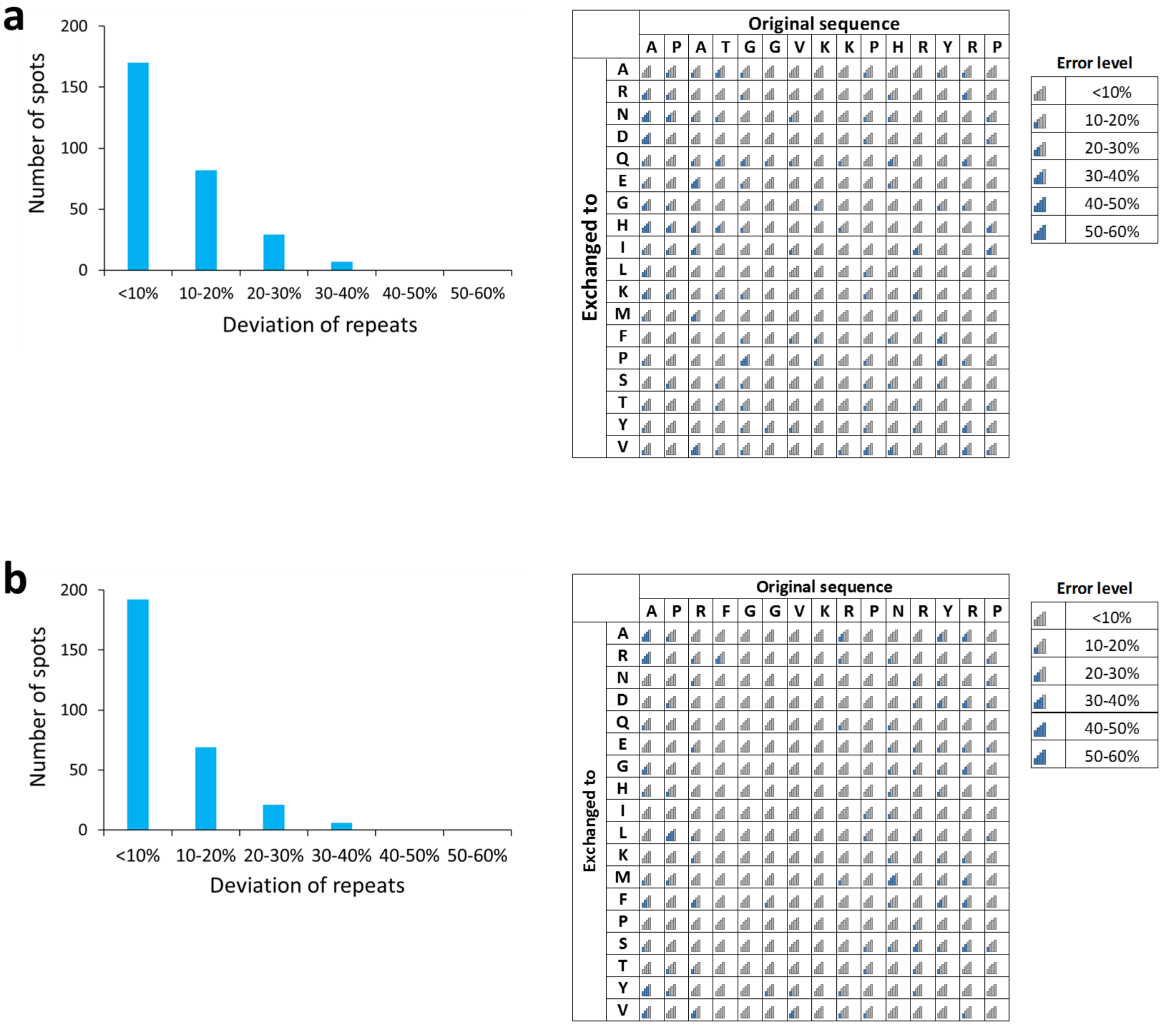

Two independent SPOT arrays were prepared and methylated by SETD2. The signals of both replicates were quantified, normalized and averaged. **a** Standard deviation of the intensities of corresponding spots in both experiments for the results in Fig. 1 a-c. **b** Standard deviation of the intensities of corresponding spots in both experiments for the results in Fig. 1 d-f. On the left side of each panel, a histogram of the error distributions is shown. On the right side, the error levels of the individual spots are displayed.

Supplementary Fig. 3: Additional kinetic analyses with the ssK36 and H3K36 peptides.

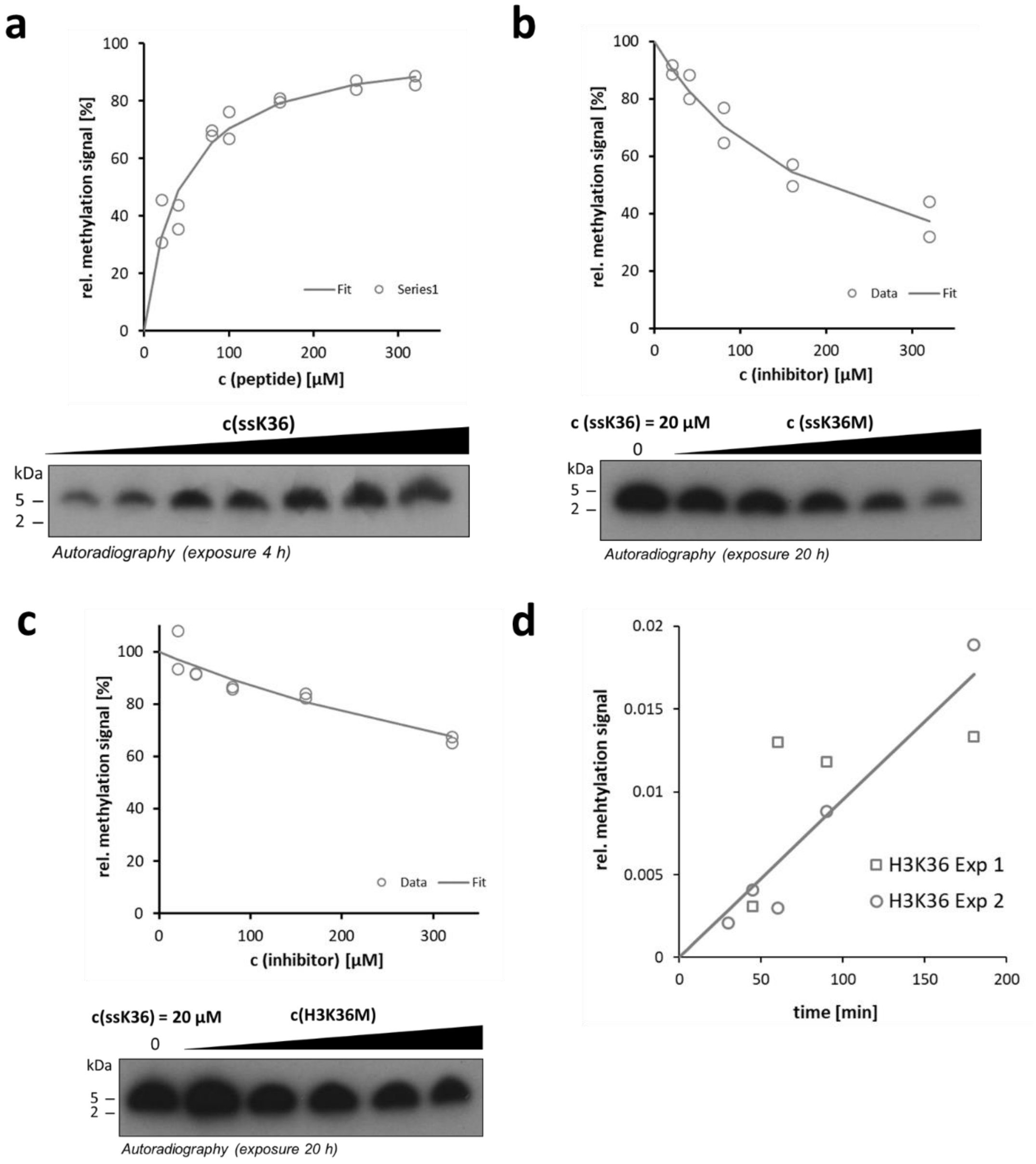

a Methylation of increasing concentration of the ssK36 peptide (20-320 µM). The data are shown as average and SD based on two independent experiments. The line shows a fit to the Michaelis-Menten model revealing a K_M of 45 µM. **b** Inhibition of ssK36 peptide (20 µM) methylation by increasing concentrations of the ssK36M inhibitor peptide. The data are shown as average and SD based on two independent experiments. The line shows a fit to a competitive inhibition model revealing a K_i of 128 µM. **c** Inhibition of ssK36 peptide (20 µM) methylation by increasing concentrations of the H3K36M inhibitor peptide. The data are shown as average and SD based on two independent experiments. The line shows a fit to a competitive inhibition model revealing a K_i of 447 µM. In panel A-C, the lower parts show one example data set. **d** Time course of methylation of H3K36 (20 µM) by SETD2 (6 µM). This panels displays an enlargement of the H3K36 data shown in Fig. 2d with adjusted y-axis.

Supplementary Fig. 4: Quantification of the data shown in Fig. 3a.

spot	A#1	A#2	spot	A#1	A#2	spot	A#1	A#2	spot	A#1	A#2	spot	A#1	A#2
A 1			C 1			E 1			G 1			I 1		
A 2			C 2			E 2			G 2			I 2		
A 3			C 3			E 3			G 3			I 3		
A 4			C 4			E 4			G 4			I 4		
A 5			C 5			E 5			G 5			I 5		
A 6			C 6			E 6			G 6			I 6		
A 7			C 7			E 7			G 7			I 7		
A 8			C 8			E 8			G 8			I 8		
A 9			C 9			E 9			G 9			I 9		
A 10			C 10			E 10			G 10			I 10		
A 11			C 11			E 11			G 11			I 11		
A 12			C 12			E 12			G 12			I 12		
A 13			C 13			E 13			G 13					
A 14			C 14			E 14			G 14					
A 15			C 15			E 15			G 15					
A 16			C 16			E 16			G 16					
A 17			C 17			E 17			G 17					
A 18			C 18			E 18			G 18					
A 19			C 19			E 19			G 19					
A 20			C 20			E 20			G 20					
B 1			D 1			F 1			H 1					
B 2			D 2			F 2			H2					
B 3			D 3			F 3			H 3					
B 4			D 4			F 4			H 4					
B 5			D 5			F 5			H 5					
B 6			D 6			F 6			H 6					
B 7			D 7			F 7			H 7					
B 8			D 8			F 8			H 8					
B 9			D 9			F 9			H 9					
B 10			D 10			F 10			H 10					
B 11			D 11			F 11			H 11					
B 12			D 12			F 12			H 12					
B 13			D 13			F 13			H 13					
B 14			D 14			F 14			H 14					
B 15			D 15			F 15			H 15					
B 16			D 16			F 16			H 16					
B 17			D 17			F 17			H 17					
B 18			D 18			F 18			H 18					
B 19			D 19			F 19			H 19					
B 20			D 20			F 20			H 20					

Quantification of two independent peptide array methylation experiments (A#1 and A#2). Light red and red cells indicate medium and strong methylation. The blue color represents known targets, grey the corresponding K-to-A mutant peptides and orange color highlights the non-histone substrates, which were further investigated in Fig. 3b.

Supplementary Fig. 5: Quantification of the data shown in Fig. 3b.

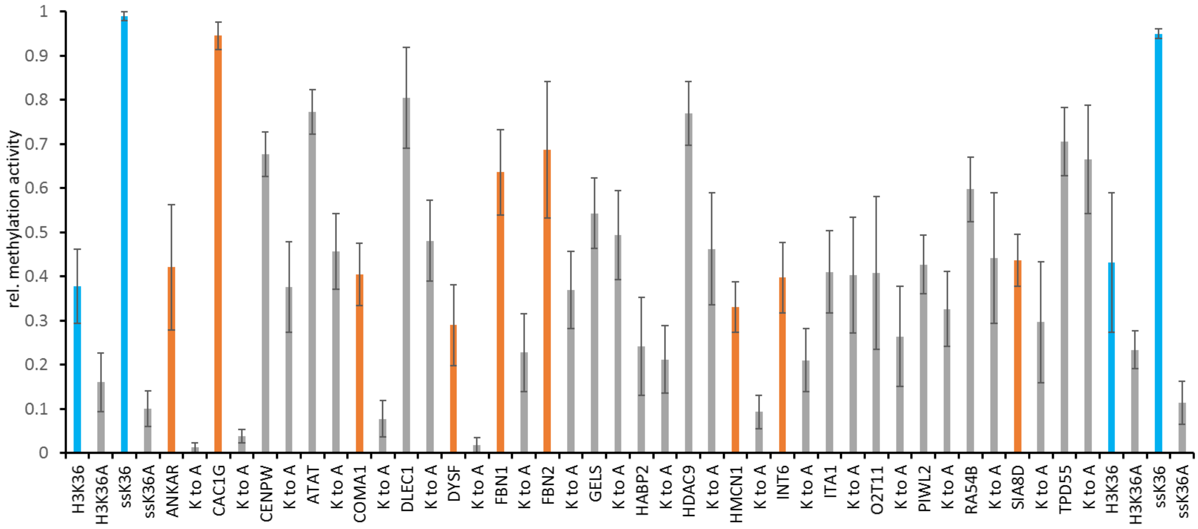

Quantification of three independent peptide array methylation experiments. The error bars indicate the standard deviation. The blue colored bars represent the positive controls and the orange colored bars highlight the non-histone substrates, which were further investigated at protein level.

Supplementary Fig. 6: Investigation of the methylation of putative non-histone protein substrates of SETD2.

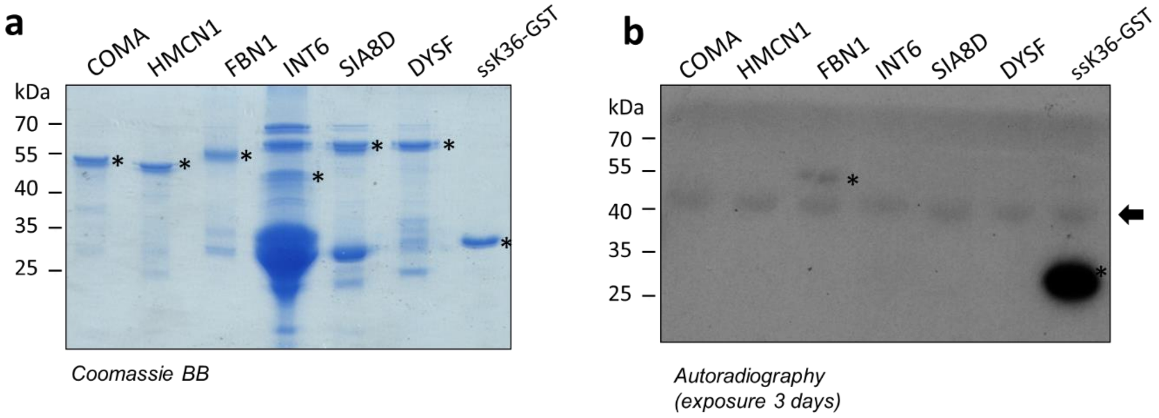

a SDS-PAGE gel of the purified candidate non-histone proteins (Table 1) and as positive control the GST tagged super-substrate (ssK36-GST). **b** Autoradiogram showing the methylation of these proteins. The arrow represents the SETD2 automethylation (50 kDa) and the black asterisk indicates the expected size of the non-histone substrate proteins and the size of the ssK36-GST protein. Methylation was only detected for the FBN1 protein and ssK36-GST.

Supplementary Fig. 7: Simulated annealing omit map with the whole peptide omitted.

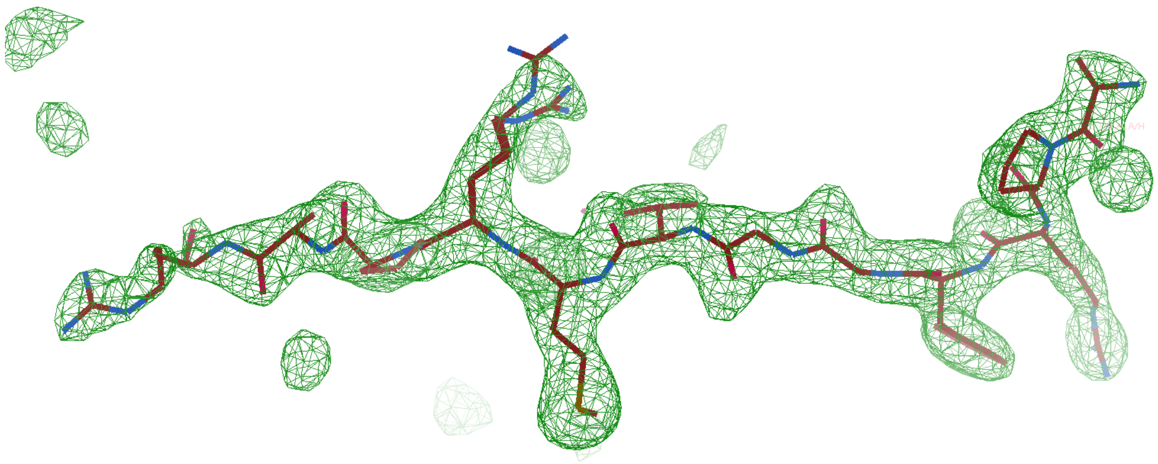

For clarity, only the histone ssK36M peptide is shown as a stick model. The mFo-DFc omit map was calculated with the Phenix software and it is shown as a green mesh contoured at the 3 rmsd level.

Supplementary Fig. 8: Hydrogen bonds and hydrophobic interactions likely to be formed between the SETD2 catalytic domain and the bound peptides.

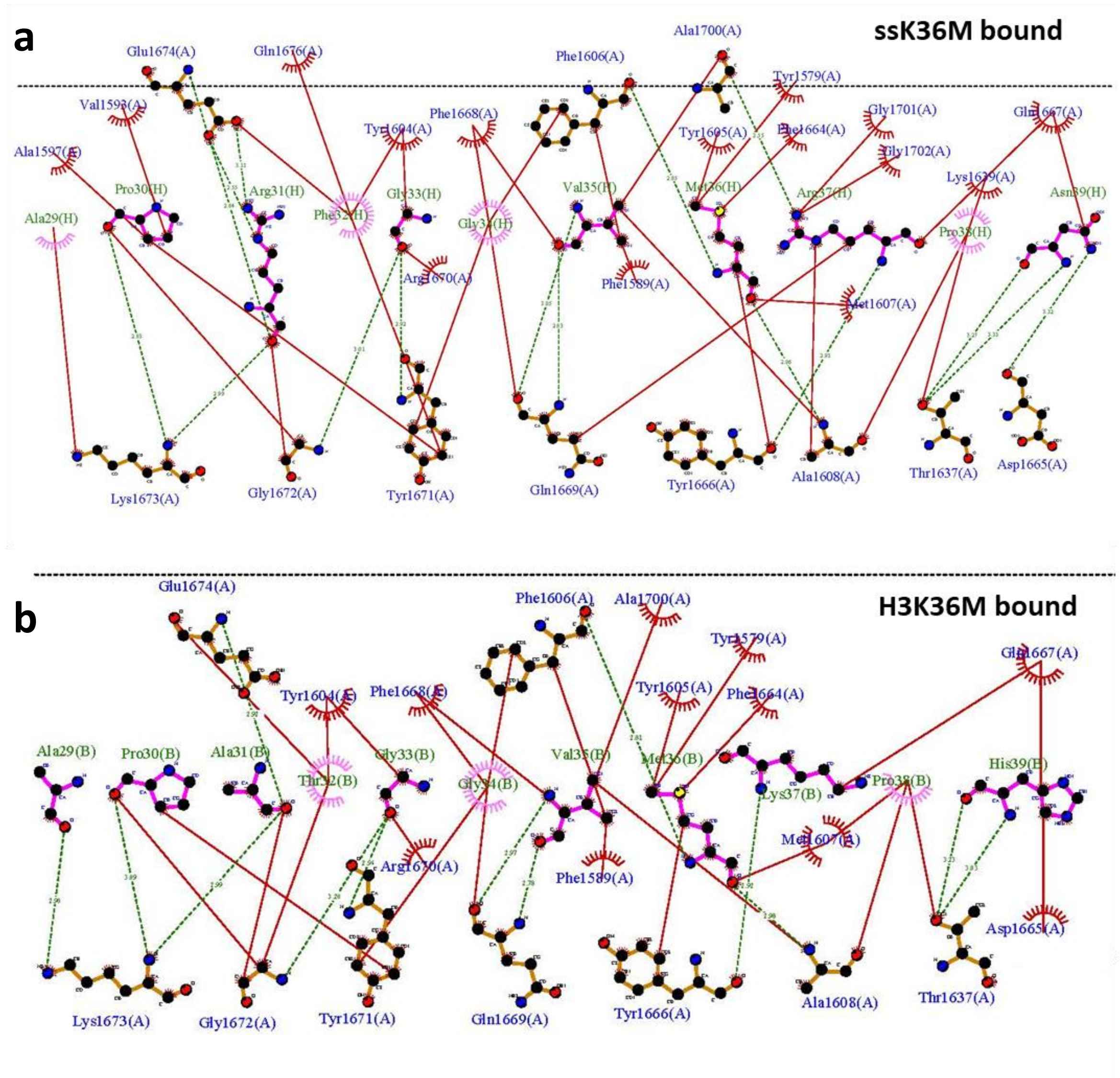

a Scheme for ssK36M. **b** Scheme for H3K36M**.** In both panels, carbon, oxygen, and nitrogen atoms are colored black, red, and blue, respectively. Green and red lines represent hydrogen bonds and hydrophobic interactions. This Fig. was generated using DIMPLOT[1] and modified.

Supplementary Fig. 9: Control experiments related to Fig. 6c.

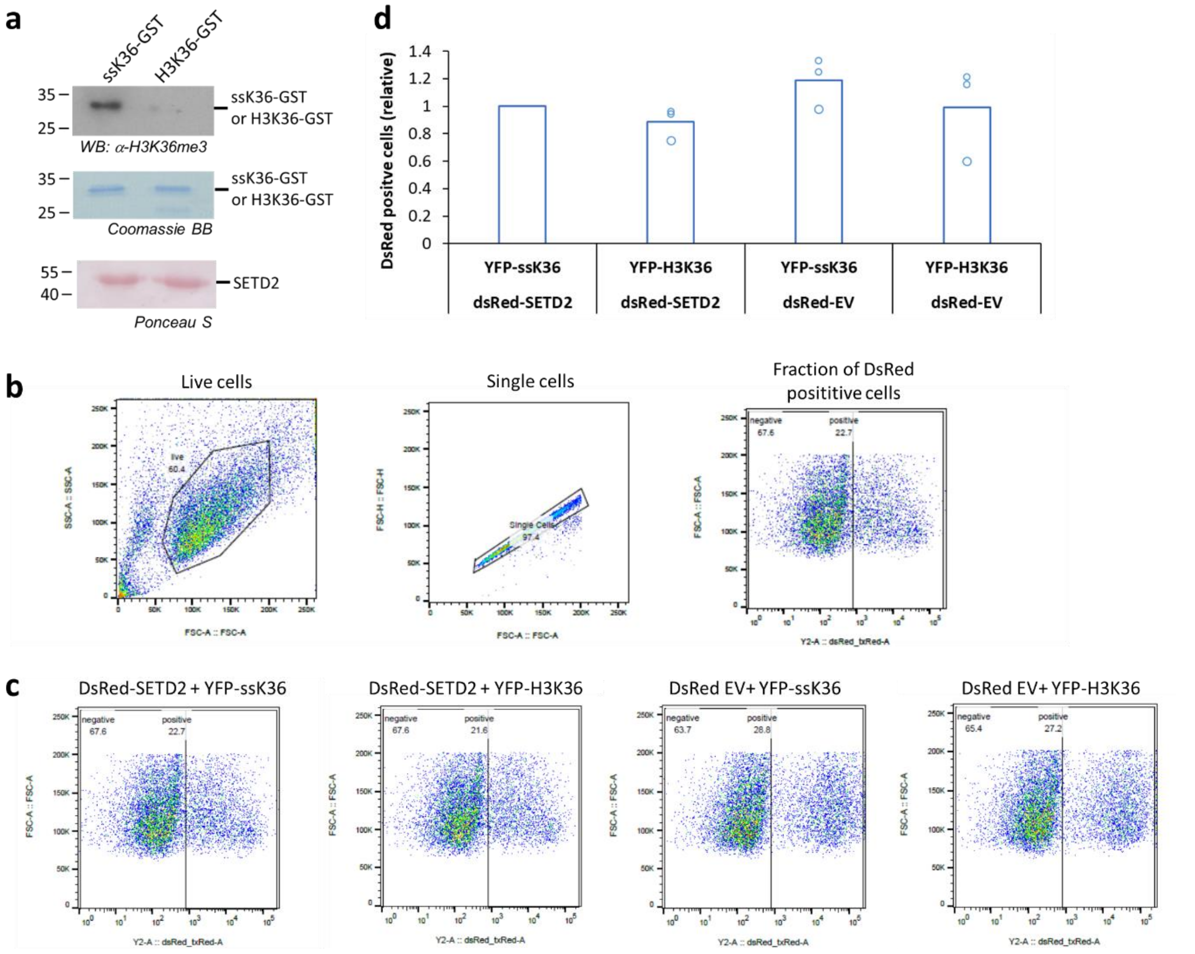

a Validation of the detection of ssK36-GST methylation by the α-H3K36me3 antibody. The purified ssK36-GST and H3K36-GST substrates were incubated with SETD2 and unlabeled AdoMet. Afterwards, the proteins were separated by SDS-PAGE and blotted. Western Blot analysis with the α-H3K36me3 antibody revealed a clear signal with ssK36-GST, while only a very weak signal was observed with H3K36-GST, which is in agreement with the very low level of H3K36-GST methylation by SETD2 observed in Fig. 6a and 6b. Equal loading of the GST-tagged proteins is shown in the Coomassie BB stain of a parallel gel, equal loading of SETD2 is shown in the Ponceau S stain of the Western Blot membrane. **b** YFP-tagged ssK36 and H3K36 substrates were transfected into HEK293 cells together with DsRed-tagged SETD2 or DsRed empty vector (EV). The expression levels of DsRed-SETD2 and DsRed from EV were determined by FACS. The Fig. shows an example of the gating strategy applied in the FACS experiments. **c** Example of the FACS primary data for DsRed expression. **d** Average fraction of DsRed positive cells in three independent experiments normalized to DsRed-SETD2 + YFP-ssK36. EV, empty vector.

Supplementary Table 1: Information related to the peptide array shown in Fig. 2a.

Spot No.	Mutation(s)	Sequence
**A 1**	-	APATGGVKKPHRYRP
**A 2**	K36A	APATGGVAKPHRYRP
**A 3**	A31R	APRTGGVKKPHRYRP
**A 4**	A31G	APGTGGVKKPHRYRP
**A 5**	T32R	APARGGVKKPHRYRP
**A 6**	T32F	APAFGGVKKPHRYRP
**A 7**	T32Y	APAYGGVKKPHRYRP
**A 8**	K37R	APATGGVKRPHRYRP
**A 9**	K37H	APATGGVKHPHRYRP
**A 10**	K37I	APATGGVKIPHRYRP
**A 11**	K37L	APATGGVKLPHRYRP
**A 12**	K37F	APATGGVKFPHRYRP
**A 13**	K37Y	APATGGVKYPHRYRP
**A 14**	K37V	APATGGVKVPHRYRP
**A 15**	H39R	APATGGVKKPRRYRP
**A 16**	H39N	APATGGVKKPNRYRP
**A 17**	H39G	APATGGVKKPGRYRP
**A 18**	H39K	APATGGVKKPKRYRP
**A 19**	H39Y	APATGGVKKPYRYRP
**A 20**	A31R, K37R	APRTGGVKRPHRYRP
**B 1**	A31G, K37R	APGTGGVKRPHRYRP
**B 2**	A31R, T32R	APRRGGVKKPHRYRP
**B 3**	A31R, T32F	APRFGGVKKPHRYRP
**B 4**	A31R, T32Y	APRYGGVKKPHRYRP
**B 5**	T32R	APARGGVKKPHRYRP
**B 6**	A31R, T32F, K37R	APRFGGVKRPHRYRP
**B 7**	A31R, T32Y, K37R	APRYGGVKRPHRYRP
**B 8**	T32R	APARGGVKKPHRYRP
**B 9**	A31R, T32F, K37Y, H39K	APRFGGVKYPKRYRP
**B 10**	A31R, T32F, K37R, H39N	APRFGGVKRPNRYRP
**B 11**	A31R, T32F, K37F	APRFGGVKFPHRYRP
**B 12**	A31R, T32F, K37Y, H39K, Y41R	APRFGGVKYPKRRRP
**B 13**	A31R, T32F, K37R, H39N, Y41R	APRFGGVKRPNRRRP
**B 14**	A31R, T32F, K37F, Y41R	APRFGGVKFPHRRRP
**B 15**	-	APATGGVKKPHRYRP
**B 16**	K36A	APATGGVAKPHRYRP

The target lysine (K36) is labeled in red and the introduced amino acid mutations are shown in blue.

Supplementary Table 2: Information related to the peptide array shown in Fig. 3a.

Spot No.	Protein name	Name	Uniprot KB No.	Target lysine	Peptide sequence	Methyl-ation
**A 1**	Histone 3.1	H3K36	P68431	36	APATGGV KKPHRYRP	+
**A 2**	Histone 3.1 K36A mutation	H3K36A		36	APATGGV AKPHRYRP	
**A 3**	Super-substrate	ssK36		36	APRFGGV KRPNRYRP	++++
**A 4**	Super-substrate K36A	ssK36A		36	APRFGGV ARPNRYRP	
**A 5**	ATP-binding cassette sub-family C member 8	ABCC8_HUMAN	Q09428	1312	ELQLGAVK RIHGLLK	
**A 6**	Acetyl-CoA carboxylase 2	ACACB_HUMAN	O00763	823	ELIYGGVK YILKVAR	
**A 7**	Neuronal acetylcholine receptor subunit alpha-5	ACHA5_HUMAN	P30532	119	PDDYGGIK VIRVPSD	
**A 8**	Acetylcholine receptor subunit alpha	ACHA_HUMAN	P02708	121	PDDYGGV KKIHIPSE	
**A 9**	Activated CDC42 kinase 1	ACK1_HUMAN	Q07912	514	PQHLGGV KKPTYDPV	
**A 10**	A-kinase anchor protein 1, mitochondrial	AKAP1_HUMAN	Q92667	800	YVDYGGYK RVKVDVL	
**A 11**	Retinal dehydrogenase 1	AL1A1_HUMAN	P00352	470	QCPFGGFK MSGNGRE	
**A 12**	Retinal dehydrogenase 2	AL1A2_HUMAN	O94788	487	QSPFGGFK MSGNGRE	
**A 13**	Aldehyde dehydrogenase family 1 member A3	AL1A3_HUMAN	P47895	481	QAPFGGF KMSGNGR E	
**A 14**	Mitochondrial 10-formyltetrahydrofolate dehydrogenase	AL1L2_HUMAN	Q3SY69	897	AAPFGGV KQSGFGK D	
**A 15**	4-trimethylaminobutyraldehyde dehydrogenase	AL9A1_HUMAN	P49189	461	ELPFGGYK KSGFGRE	
**A 16**		AL9A1_HUMAN	P49189	344	ERVLGFVK VAKEQGA	
**A 17**	Aldehyde dehydrogenase, mitochondrial	ALDH2_HUMAN	P05091	486	QSPFGGYK MSGSGRE	
**A 18**	Ankyrin and armadillo repeat-containing protein	ANKAR_HUMAN	Q7Z5J8	317	RRGIGYLK LICFLIP	++
**A 19**	Progressive ankylosis protein homolog	ANKH_HUMAN	Q9HCJ1	214	TLCLGYYK NIHDIIP	
**A 20**	Protein arginine N-methyltransferase 3	ANM3_HUMAN	O60678	91	LEFYGYIKLI NFIRL	
**B 1**	MICOS complex subunit MIC27	MIC27_HUMAN	Q6UXV4	180	QQIFGAVK SLWTKSS	
**B 2**	Advillin	AVIL_HUMAN	O75366	109	DTFRGYFK QGIIYKQ	

**B 3**	BAH and coiled-coil domain-containing protein 1	BAHC1_HUMAN	Q9P281	659	LVGLGGLKASCIQQE	
**B 4**	Bromodomain adjacent to zinc finger domain protein 1A	BAZ1A_HUMAN	Q9NRL2	999	QGTLGAIKVTDRHIW	
**B 5**	Betaine-homocysteine S-methyltransferase 1	BHMT1_HUMAN	Q93088	40	LEKRGYVKAGPWTPE	
**B 6**	S-methylmethionine--homocysteine S-methyltransferase BHMT2	BHMT2_HUMAN	Q9H2M3	40	LEKRGYVKAGLWTPE	
**B 7**	Ankyrin repeat and BTB/POZ domain-containing protein BTBD11	BTBDB_HUMAN	A6QL63	969	CIEIGYVKYSIFQLV	
**B 8**	MHC class II transactivator	C2TA_HUMAN	P33076	1034	ITDLGAYKLAEALPS	
**B 9**	SprT-like domain-containing protein Spartan	SPRTN_HUMAN	Q9H040	184	PPYYGYVKRATNREP	
**B 10**	Voltage-dependent T-type calcium channel subunit alpha-1G	CAC1G_HUMAN	O43497	804	YGPFGYIKNPYNIFD	++
**B 11**	UPF0565 protein C2orf69	CB069_HUMAN	Q8N8R5	185	NTDFGAFKHLYMLLV	
**B 12**	Cyclin-dependent kinase 3	CDK3_HUMAN	Q00526	142	INELGAIKLADFGLA	
**B 13**	Carcinoembryonic antigen-related cell adhesion molecule 7	CEAM7_HUMAN	Q14002	84	YRIIGYVKNISQENA	
**B 14**	Centromere protein W	CENPW_HUMAN	Q5EE01	23	KAPRGFLKRVFKRKK	++
**B 15**	Alpha-tubulin N-acetyltransferase 1	ATAT_HUMAN	Q5SQI0	98	GAIIGFIKVGYKKLF	+++
**B 16**	UPF0553 protein C9orf64	CI064_HUMAN	Q5T6V5	253	LAHLGALKYSDDLLK	
**B 17**	Cilia- and flagella-associated protein 77	CFA77_HUMAN	Q6ZQR2	165	AMNRGAVKAGLVTAR	
**B 18**	Uncharacterized protein C10orf67, mitochondrial	CJ067_HUMAN	Q8IYJ2	123	QVDFGFLKQLLQLKF	
**B 19**	Uncharacterized protein C11orf65	CK065_HUMAN	Q8NCR3	78	RFRLGGVKFPPDIYY	
**B 20**	Transmembrane protein 260	TM260_HUMAN	Q9NX78	692	ADILGALKHLRKELQ	
**C 1**	Collagen alpha-3(VI) chain	CO6A3_HUMAN	P12111	310	AQVLGAVKALGFAGG	
**C 2**	Collagen alpha-5(VI) chain	CO6A5_HUMAN	A8TX70	857	RVQFGALKYSDQPNI	
**C 3**	Collagen alpha-6(VI) chain	CO6A6_HUMAN	A6NMZ7	852	QVRFGALKYADDPEV	

**C 4**	Collagen alpha-1(XIV) chain	COEA1_HUMAN	Q05707	61	RGKFGGYKLLVTPTS	
**C 5**	Conserved oligomeric Golgi complex subunit 2	COG2_HUMAN	Q14746	584	DSCFGFLKSALEVPR	
**C 6**	Collagen alpha-1(XXII) chain	COMA1_HUMAN	Q8NFW1	472	SEQIGFLKTINCSCP	++
**C 7**	Protein FAM189B	F189B_HUMAN	P81408	171	DEARGALKNLLFSVC	
**C 8**	Cytochrome P450 4B1	CP4B1_HUMAN	P13584	28	ILVLGFLKLIHLLLR	
**C 9**	Uncharacterized protein KIAA0930	K0930_HUMAN	Q6ICG6	236	KMSFGFYKYSNMEFV	
**C 10**	ATP-dependent RNA helicase DDX54	DDX54_HUMAN	Q8TDD1	848	FLQRGGLKQLSARNR	
**C 11**	Deleted in lung and esophageal cancer protein 1	DLEC1_HUMAN	Q9Y238	494	YCLIGGVKMTRFICK	+++
**C 12**	Dual specificity phosphatase DUPD1	DUPD1_HUMAN	Q68J44	191	LPNRGFLKQLRELDK	
**C 13**	Integrator complex subunit 6-like	INTL6_HUMAN	Q5JSJ4	373	GYPFGYLKASTTLTC	+
**C 14**	Dynein heavy chain 1, axonemal	DYH1_HUMAN	Q9P2D7	2332	RKIIGAFKNLVDINF	
**C 15**	Dysferlin	DYSF_HUMAN	O75923	338	AGARGYLKTSLCVLG	++
**C 16**	Endonuclease/exonuclease/phosphatase family domain-containing protein 1	EEPD1_HUMAN	Q7L9B9	75	REYIGGFKKVEDLAL	
**C 17**	Ephrin type-A receptor 4	EPHA4_HUMAN	P54764	282	ACKIGYYKALSTDAT	
**C 18**	Ephrin type-A receptor 7	EPHA7_HUMAN	Q15375	285	PCGRGFYKSSSQDLQ	
**C 19**	Ephrin type-A receptor 8	EPHA8_HUMAN	P29322	282	ACELGFYKSAPGDQL	
**C 20**	FAST kinase domain-containing protein 5, mitochondrial	FAKD5_HUMAN	Q7L8L6	327	TICLGFFKSSTNLSE	
**D 1**	Fibrillin-1	FBN1_HUMAN	P35555	666	STCYGGYKRGQCIKP	+++
**D 2**	Fibrillin-2	FBN2_HUMAN	P35556	711	STCYGGIKKGVCVRP	+++
**D 3**	Fidgetin-like protein 1	FIGL1_HUMAN	Q6PIW4	325	GSSYGGVKKSLGASR	
**D 4**	Cytosolic 10-formyltetrahydrofolate dehydrogenase	AL1L1_HUMAN	O75891	876	AAPFGGFKQSGFGKD	
**D 5**	FYN-binding protein	FYB_HUMAN	O15117	563	RGSYGYIKTTAVEID	
**D 6**	Endogenous retrovirus group K member 10 Gag polyprotein	GAK10_HUMAN	P87889	29	LLKRGGVKVSTKNLI	+

**D 7**	Endogenous retrovirus group K member 5 Gag polyprotein	GAK5_HUMAN	Q9HDB9	555	CGQIGHLKRSCPVLN	+
**D 8**	Polypeptide N-acetylgalactosaminyltransferase 6	GALT6_HUMAN	Q8NCL4	502	PTFYGAIKNLGTNQC	+
**D 9**	Growth arrest-specific protein 6	GAS6_HUMAN	Q14393	269	CDGRGGLKLSQDMDT	
**D 10**	eIF-2-alpha kinase activator GCN1	GCN1L_HUMAN	Q92616	599	LSSLGGFKLAHGLLE	
**D 11**	Gelsolin	GELS_HUMAN	P06396	162	ATFLGYFKSGLKYKK	+++
**D 12**	Glucosamine 6-phosphate N-acetyltransferase	GNA1_HUMAN	Q96EK6	55	DLNRGFFKVLGQLTE	
**D 13**	Solute carrier family 2, facilitated glucose transporter member 10	GTR10_HUMAN	O95528	280	SVGLGAVKVAATLTA	
**D 14**	Histone H4	H4_HUMAN	P62805	45	LARRGGVKRISGLIY	
**D 15**	Hyaluronan-binding protein 2	HABP2_HUMAN	Q14520	319	KRIYGGFKSTAGKHP	++
**D 16**	Histone deacetylase 9	HDAC9_HUMAN	Q9UKV0	927	TPPLGGYKVTAKCFG	+++
**D 17**	Hemicentin-1	HMCN1_HUMAN	Q96RW7	127	EMSIGAIKIALEISL	
**D 18**	Hemicentin-1	HMCN1_HUMAN	Q96RW7	5253	KNTRGGYKCIDLCPN	++
**D 19**	Insulin receptor-related protein	INSRR_HUMAN	P14616	397	LVSLGFFKNLKLIRG	
**D 20**	Integrator complex subunit 6	INT6_HUMAN	Q9UL03	369	GHPFGYLKASTALNC	++
**E 1**	Integrator complex subunit 6	INT6_HUMAN	Q9UL03	428	PYYLGPLKKAVRMMG	
**E 2**	IQ domain-containing protein G	IQCG_HUMAN	Q9H095	417	RREIGGFKMPKDKVD	
**E 3**	Integrin alpha-1	ITA1_HUMAN	P56199	1170	LWKIGFFKRPLKKKM	++
**E 4**	Integrin alpha-5	ITA5_HUMAN	P08648	1027	LYKLGFFKRSLPYGT	
**E 5**	Integrin alpha-9	ITA9_HUMAN	Q13797	653	YLALGAVKNISLNIS	
**E 6**	Tectonin beta-propeller repeat-containing protein 2	TCPR2_HUMAN	O15040	1217	LSQLGAVKLTSLACG	+
**E 7**	TELO2-interacting protein 1 homolog	TTI1_HUMAN	O43156	409	SLLLGYLKLLGPKIN	
**E 8**	Little elongation complex subunit 1	ICE1_HUMAN	Q9Y2F5	787	KSGLGFVKSTSWHHS	+

**E 9**	Protein kinase C delta type	KPCD_HUMAN	Q05655	149	MNRRGAIKQAKIHYI	+
**E 10**	Protein kinase C theta type	KPCT_HUMAN	Q04759	150	HQRRGAIKQAKVHHV	+
**E 11**	Pyruvate kinase PKM	KPYM_HUMAN	P14618	504	GKARGFFKKGDVVIV	
**E 12**	LIM and senescent cell antigen-like-containing domain protein 1	LIMS1_HUMAN	P48059	111	LADIGFVKNAGRHLC	+
**E 13**	LIM and senescent cell antigen-like-containing domain protein 2	LIMS2_HUMAN	Q7Z4I7	116	LADLGFVKNAGRHLC	+
**E 14**	Leiomodin-1	LMOD1_HUMAN	P29536	126	EPKRGGLKKSFSRDR	
**E 15**	Protein phosphatase 1 regulatory subunit 37	PPR37_HUMAN	O75864	81	DEVIGAYKQACQKLN	
**E 16**	Leucine-rich repeats and immunoglobulin-like domains protein 3	LRIG3_HUMAN	Q6UXM1	242	FQGLGALKSLKMQRN	
**E 17**	Low-density lipoprotein receptor-related protein 2	LRP2_HUMAN	P98164	4116	GSRFGAIKRAYIPNF	
**E 18**	MAM domain-containing protein 2	MAMC2_HUMAN	Q7Z304	423	YAIYGFLKMSDTLAV	
**E 19**	Multiple epidermal growth factor-like domains protein 8	MEGF8_HUMAN	Q7Z7M0	941	GRGRGALKSPEECPP	
**E 20**	Mitoferrin-1	MFRN1_HUMAN	Q9NYZ2	92	TSIYGALKKIMRTEG	
**F 1**	Putative helicase MOV-10	MOV10_HUMAN	Q9HCE1	900	DFNLGFLKNPKRFNV	
**F 2**	Metastasis-associated protein MTA1	MTA1_HUMAN	Q13330	431	WKKYGGLKMPTRLDG	
**F 3**	Metastasis-associated protein MTA2	MTA2_HUMAN	O94776	405	WKKYGGLKTPTQLEG	
**F 4**	Metastasis-associated protein MTA3	MTA3_HUMAN	Q9BTC8	417	WKKYGGLKMPTQSEE	
**F 5**	Myocardin	MYCD_HUMAN	Q8IZQ8	644	HSPLGAVKSPQHISL	
**F 6**	N-acetyl-D-glucosamine kinase	NAGK_HUMAN	Q9UJ70	185	PHDIGYVKQAMFHYF	
**F 7**	Neuroblastoma-amplified sequence	NBAS_HUMAN	A2RRP1	490	ARYFGYIKQGLYLVT	
**F 8**	Nuclear factor of activated T-cells, cytoplasmic 1	NFAC1_HUMAN	O95644	452	EGSRGAVKASAGGHP	
**F 9**	Nuclear factor of activated T-cells, cytoplasmic 2	NFAC2_HUMAN	Q13469	434	EGSRGAVKAPTGGHP	

**F 10**	Nuclear factor of activated T-cells, cytoplasmic 3	NFAC3_HUMAN	Q12968	457	EGSRGAVKASTGGHP	
**F 11**	Nuclear factor of activated T-cells, cytoplasmic 4	NFAC4_HUMAN	Q14934	443	EGSRGAVKAAPGGHP	
**F 12**	Nucleotide-binding oligomerization domain-containing protein 1	NOD1_HUMAN	Q9Y239	633	SSLRGYLKSLPRVQV	
**F 13**	Olfactory receptor 2T11	O2T11_HUMAN	Q8NH01	299	KDVIGAFKKVFACCS	+++
**F 14**	Obscurin	OBSCN_HUMAN	Q5VST9	6483	RGVFGFVKRVQHKGN	
**F 15**	Olfactory receptor 2J1	OR2J1_HUMAN	Q9GZK6	303	KDVRGAVKRLMGWEW	
**F 16**	Olfactory receptor 2J3	OR2J3_HUMAN	O76001	305	NKVVRGAVKRLMGWE	
**F 17**	Tumor protein p73	P73_HUMAN	O15350	532	IEDLGALKIPEQYRM	
**F 18**	Procollagen C-endopeptidase enhancer 1	PCOC1_HUMAN	Q15113	365	VSLIGAYKTGGLDLP	
**F 19**	Pecanex-like protein 3	PCX3_HUMAN	Q9H6A9	973	GFCLGAIKTPWPEQH	
**F 20**	High affinity cGMP-specific 3',5'-cyclic phosphodiesterase 9A	PDE9A_HUMAN	O76083	519	TAQIGFIKFVLIPMF	+
**G 1**	PDZ domain-containing protein 8	PDZD8_HUMAN	Q8NEN9	423	LIAIGGVKITSTLQV	
**G 2**	Phosphate-regulating neutral endopeptidase	PHEX_HUMAN	P78562	319	FDWLGYIKKVIDTRL	
**G 3**	Piwi-like protein 2	PIWL2_HUMAN	Q8TC59	685	DDLYGAIKKLCCVQS	+++
**G 4**	Plakophilin-4	PKP4_HUMAN	Q99569	573	VCRLGGIKHLVDLLD	+
**G 5**	Putative PRAME family member 13	PRA13_HUMAN	Q5VWM6	323	YPSLGYLKHLNLSYV	
**G 6**	PRAME family member 2	PRAM2_HUMAN	O60811	323	FPSLGYLKHLNLSYV	
**G 7**	Receptor-type tyrosine-protein phosphatase gamma	PTPRG_HUMAN	P23470	1093	VNVLGFLKHIRTQRN	
**G 8**	Receptor-type tyrosine-protein phosphatase zeta	PTPRZ_HUMAN	P23471	1966	VNIFGFLKHIRSQRN	
**G 9**	DNA repair and recombination protein RAD54B	RA54B_HUMAN	Q9Y620	589	LICIGALKKLCNHPC	+++
**G 10**	Ras-related C3 botulinum toxin substrate 1	RAC1_HUMAN	P63000	153	AKEIGAVKYLECSAL	

**G 11**	E3 ubiquitin-protein ligase RBBP6	RBBP6_HUMAN	Q7Z6E9	81	RIPIGGVKSTSKTYV	+
**G 12**	Rho GTPase-activating protein 1	RHG01_HUMAN	Q07960	109	SKLLGYLKHTLDQYV	
**G 13**	Rho GTPase-activating protein 6	RHG06_HUMAN	O43182	132	PLGRGGLKKSMAWDL	
**G 14**	RING finger protein 214	RN214_HUMAN	Q8ND24	541	PPGLGGVKASAETPR	
**G 15**	Oxygen-regulated protein 1	RP1_HUMAN	P56715	1629	EYNIGFVKRAIEKLY	
**G 16**	RRP12-like protein	RRP12_HUMAN	Q5JTH9	977	KSALGFIKVAVTVMD	
**G 17**	Protein RRP5 homolog	RRP5_HUMAN	Q14690	737	MLLIGFVKSIKDYGV	
**G 18**	Sodium-dependent multivitamin transporter	SC5A6_HUMAN	Q9Y289	197	YTALGGLKAVIWTDV	
**G 19**	Sodium-coupled monocarboxylate transporter 1	SC5A8_HUMAN	Q8N695	183	YCTLGGLKAVIWTDV	
**G 20**	Sodium-coupled monocarboxylate transporter 2	SC5AC_HUMAN	Q1EHB4	179	YCTLGGLKAVVWTDA	
**H 1**	Sodium channel protein type 1 subunit alpha	SCN1A_HUMAN	P35498	1313	YSELGAIKSLRTLRA	
**H 2**	Sodium channel protein type 4 subunit alpha	SCN4A_HUMAN	P35499	126	VVRRGAIKVLIHALF	
**H 3**	Sodium channel protein type 8 subunit alpha	SCN8A_HUMAN	Q9UQD0	1258	WTAYGFVKFFTNAWC	
**H 4**	Splicing factor 3B subunit 1	SF3B1_HUMAN	O75533	998	GSILGALKAIVNVIG	
**H 5**	Epsilon-sarcoglycan	SGCE_HUMAN	O43556	188	GDFLGAVKNVWQPER	
**H 6**	CMP-N-acetylneuraminate-poly-alpha-2,8-sialyltransferase	SIA8D_HUMAN	Q92187	350	LHNRGALKLTTGKCV	+++
**H 7**	Succinate-semialdehyde dehydrogenase, mitochondrial	SSDH_HUMAN	P51649	508	ECPFGGVKQSGLGRE	
**H 8**	Symplekin	SYMPK_HUMAN	Q92797	567	AMKLGAVKRILRAEK	
**H 9**	Synaptophysin	SYPH_HUMAN	P08247	30	KEPLGFVKVLQWVFA	
**H 10**	Synaptophysin-like protein 1	SYPL1_HUMAN	Q16563	37	KEPLGFIKVLEWIAS	
**H 11**	Synaptophysin-like protein 2	SYPL2_HUMAN	Q5VXT5	39	EEPLGFIKVLQWLFA	
**H 12**	Threonine--tRNA ligase, mitochondrial	SYTM_HUMAN	Q9BW92	255	TGQIGGLKLLSNSSS	
**H 13**	Taste receptor type 2 member 40	T2R40_HUMAN	P59535	247	KAHIGAIKATSYFLI	

**H 14**	Putative ATP-dependent RNA helicase TDRD12	TDR12_HUMAN	Q587J7	884	LPSFGYIKIIPFYIL	
**H 15**	Transmembrane protein 14C	TM14C_HUMAN	Q9P0S9	31	GGIIGYVKAGSVPSL	
**H 16**	Tumor necrosis factor ligand superfamily member 13B	TN13B_HUMAN	Q9Y275	283	SLDGDVTFFGALKLL	
**H 17**	Tumor protein D55	TPD55_HUMAN	Q96J77	112	CRKLGGVKKSATFRS	+++
**H 18**	tRNA selenocysteine 1-associated protein 1	TSAP1_HUMAN	Q9NX07	143	SKGYGFVKFTDELEQ	+
**H 19**	UDP-N-acetylhexosamine pyrophosphorylase-like protein 1	UAP1L_HUMAN	Q3KQV9	342	FFTRGFLKAVTREFE	
**H 20**	Ubiquitin-like modifier-activating enzyme 1	UBA1_HUMAN	P22314	97	NIILGGVKAVTLHDQ	
**I 1**	Ubiquitin-like protein 3	UBL3_HUMAN	O95164	77	NVTLGALKLPFGKTT	
**I 2**	Ubiquitin carboxyl-terminal hydrolase 40	UBP40_HUMAN	Q9NVE5	933	LPPLGFLKVPIWWYQ	
**I 3**	Ubiquitin carboxyl-terminal hydrolase 47	UBP47_HUMAN	Q96K76	688	GLLLGGVKSTYMFDL	
**I 4**	Villin-1	VILI_HUMAN	P09327	112	EAFRGYFKQGLVIRK	
**I 5**	Serine/threonine-protein kinase WNK3	WNK3_HUMAN	Q9BYP7	159	ELGRGAFKTVYKGLD	
**I 6**	Zinc finger CCCH domain-containing protein 7B	Z3H7B_HUMAN	Q9UGR2	549	FDPLGGVKRGSLTIA	
**I 7**	Palmitoyltransferase ZDHHC15	ZDH15_HUMAN	Q96MV8	268	GFNLGFIKNIQQVFG	
**I 8**	Palmitoyltransferase ZDHHC5	ZDHC5_HUMAN	Q9C0B5	701	SPTRGGVKKVSGVGG	
**I 9**	Histone 3.1	H3K36	P68431	36	APTAGGVKKPHRYRP	+
**I 10**	Histone 3.1 A mutation	H3K36A		36	APATGGVAKPHRYRP	
**I 11**	Super-substrate	ssK36		36	APRFGGVKRPNRYRP	++++
**I 12**	Super-substrate K36A	ssK36A		36	APRFGGVARPNRYRP	

The predicted target lysine is shown in red. The H3K36 (29-43) sequence and the sequence of the ssK36 peptide were used as positive controls. As negative controls, their respective lysine to alanine mutations were included.

Supplementary Table 3: Information related to the peptide array shown in Fig. 3b.

Spot No.	Protein name	Name	Uniprot KB No.	Target lysine	Peptide sequence
**A 1**	Histone H3.1	H3K36	P68431	36	APATGGVKKPHRYRP
**A 2**					APATGGVAKPHRYRP
**A 3**	Super-substrate	ssK36		8	APRFGGVKRPNRYRP
**A 4**					APRFGGVARPNRYRP
**A 5**	Ankyrin and armadillo repeat-containing protein	ANKAR_HUMAN	Q7Z5J8	317	RRGIGYLKLICFLIP
**A 6**					RRGIGYLALICFLIP
**A 7**	Voltage-dependent T-type calcium channel subunit alpha-1G	CAC1G_HUMAN	O43497	804	YGPFGYIKNPYNIFD
**A 8**					YGPFGYIANPYNIFD
**A 9**	Centromere protein W	CENPW_HUMAN	Q5EE01	23	KAPRGFLKRVFKRKK
**A 10**					KAPRGFLARVFKRKK
**A 11**	Alpha-tubulin N-acetyltransferase 1	ATAT_HUMAN	Q5SQI0	98	GAIIGFIKVGYKKLF
**A 12**					GAIIGFIAVGYKKLF
**A 13**	Collagen alpha-1(XXII) chain	COMA1_HUMAN	Q8NFW1	472	SEQIGFLKTINCSCP
**A 14**					SEQIGFLATINCSCP
**A 15**	Deleted in lung and esophageal cancer protein 1	DLEC1_HUMAN	Q9Y238	494	YCLIGGVKMTRFICK
**A 16**					YCLIGGVAMTRFICK
**A 17**	Dysferlin	DYSF_HUMAN	O75923	338	AGARGYLKTSLCVLG
**A 18**					AGARGYLATSLCVLG
**A 19**	Fibrillin-1	FBN1_HUMAN	P35555	666	STCYGGYKRGQCIKP
**A 20**					STCYGGYARGQCIKP
**B 1**	Fibrillin-2	FBN2_HUMAN	P35556	711	STCYGGIKKGVCVRP
**B 2**					STCYGGIAKGVCVRP
**B 3**	Gelsolin	GELS_HUMAN	P06396	162	ATFLGYFKSGLKYKK
**B 4**					ATFLGYFASGLKYKK
**B 5**	Hyaluronan-binding protein 2	HABP2_HUMAN	Q14520	319	KRIYGGFKSTAGKHP
**B 6**					KRIYGGFASTAGKHP
**B 7**	Histone deacetylase 9	HDAC9_HUMAN	Q9UKV0	927	TPPLGGYKVTAKCFG

**B 8**					TPPLGGYAVTAKCFG
**B 9**	Hemicentin-1	HMCN1_HUMAN	Q96RW7	127	EMSIGAIKIALEISL
**B 10**					EMSIGAIAIALEISL
**B 11**	Integrator complex subunit 6	INT6_HUMAN	Q9UL03	369	GHPFGYLKASTALNC
**B 12**					GHPFGYLAASTALNC
**B 13**	Integrin alpha-1	ITA1_HUMAN	P56199	1170	LWKIGFFKRPLKKKM
**B 14**					LWKIGFFARPLKKKM
**B 15**	Olfactory receptor 2T11	O2T11_HUMAN	Q8NH01	299	KDVIGAFKKVFACCS
**B 16**					KDVIGAFAKVFACCS
**B 17**	Piwi-like protein 2	PIWL2_HUMAN	Q8TC59	685	DDLYGAIKKLCCVQS
**B 18**					DDLYGAIAKLCCVQS
**B 19**	DNA repair and recombination protein RAD54B	RA54B_HUMAN	Q9Y620	589	LICIGALKKLCNHPC
**B 20**					LICIGALAKLCNHPC
**C 1**	CMP-N-acetylneuraminate-poly-alpha-2,8-sialyltransferase	SIA8D_HUMAN	Q92187	350	LHNRGALKLTTGKCV
**C 2**					LHNRGALALTTGKCV
**C 3**	Tumor protein D55	TPD55_HUMAN	Q96J77	112	CRKLGGVKKSATFRS
**C 4**					CRKLGGVAKSATFRS
**C 5**	Histone H3.1	H3K36	P68431	36	APATGGVKKPHRYRP
**C 6**					APATGGVAKPHRYRP
**C 7**	Super-substrate	ssK36		36	APRFGGVKRPNRYRP
**C 8**					APRFGGVARPNRYRP

The predicted target lysine is shown in red and the corresponding alanine mutant in blue. The positive controls H3K36 (29-43) and the ssK36 peptide and their respective lysine to alanine mutations as negative controls were included as well.

Supplementary references

1 Laskowski, R. A. & Swindells, M. B. LigPlot+: multiple ligand-protein interaction diagrams for drug discovery. *Journal of chemical information and modeling* **51**, 2778-2786 (2011).
