# Supplementary material for: Sequence specificity analysis of the SETD2 protein lysine methyltransferase and discovery of a SETD2 super-substrate
Source: Commun Biol. 2020 Sep 16;3:511. doi: 10.1038/s42003-020-01223-6 (PMC7495481; doi:10.1038/s42003-020-01223-6)
Supplement: Supplementary file 2 — Description of Additional Supplementary Files [file 42003_2020_1223_MOESM2_ESM.docx]

Description of Additional Supplementary Files

Supplementary Data 1: Source data underlying plots shown in figures and full blots.
