# Supplementary material for: Sequence specificity analysis of the SETD2 protein lysine methyltransferase and discovery of a SETD2 super-substrate
Source: Commun Biol. 2020 Sep 16;3:511. doi: 10.1038/s42003-020-01223-6 (PMC7495481; doi:10.1038/s42003-020-01223-6)
Supplement: Supplementary file 4 — Reporting Summary [file 42003_2020_1223_MOESM4_ESM.pdf]

nature research

Corresponding author(s): Albert Jeltsch, Jinrong Min

Last updated by author(s): Jul 19, 2020

Reporting Summary

Nature Research wishes to improve the reproducibility of the work that we publish. This form provides structure for consistency and transparency in reporting. For further information on Nature Research policies, see Authors & Referees and the Editorial Policy Checklist.

Statistics

For all statistical analyses, confirm that the following items are present in the figure legend, table legend, main text, or Methods section.

n/a	Confirmed	
☐	☒	The exact sample size (n) for each experimental group/condition, given as a discrete number and unit of measurement
☐	☒	A statement on whether measurements were taken from distinct samples or whether the same sample was measured repeatedly
☐	☒	The statistical test(s) used AND whether they are one- or two-sided<br>*Only common tests should be described solely by name; describe more complex techniques in the Methods section.*
☒	☐	A description of all covariates tested
☒	☐	A description of any assumptions or corrections, such as tests of normality and adjustment for multiple comparisons
☒	☐	A full description of the statistical parameters including central tendency (e.g. means) or other basic estimates (e.g. regression coefficient) AND variation (e.g. standard deviation) or associated estimates of uncertainty (e.g. confidence intervals)
☒	☐	For null hypothesis testing, the test statistic (e.g. F, t, r) with confidence intervals, effect sizes, degrees of freedom and P value noted<br>*Give P values as exact values whenever suitable.*
☒	☐	For Bayesian analysis, information on the choice of priors and Markov chain Monte Carlo settings
☒	☐	For hierarchical and complex designs, identification of the appropriate level for tests and full reporting of outcomes
☒	☐	Estimates of effect sizes (e.g. Cohen's d, Pearson's r), indicating how they were calculated

Our web collection on statistics for biologists contains articles on many of the points above.

Software and code

Policy information about availability of computer code

Data collection	Diffraction experiments were conducted initially on a rotating copper anode and, later, at Advanced Photon Source beam line 24-ID-E.
Data analysis	Diffraction images were processed with XDS and AIMLESS. Coordinates from isomorphous PDB entry 5JLB chain A were refined against these diffraction data using DIMPLE/REFMAC. The model was interactively rebuilt in COOT and further refined in REFMAC, AUTOBUSTER (Bricogne G., et al., 2017, BUSTER. Cambridge, United Kingdom: Global Phasing Ltd.) and PHENIX. The latest round of refinement was performed with BUSTER against the weaker, but earlier collected, copper anode data, which we preferred due to its lower radiation load and concerns about radiation-induced decarboxylation of glutamate residues.

For manuscripts utilizing custom algorithms or software that are central to the research but not yet described in published literature, software must be made available to editors/reviewers. We strongly encourage code deposition in a community repository (e.g. GitHub). See the Nature Research guidelines for submitting code & software for further information.

Data

Policy information about availability of data

All manuscripts must include a data availability statement. This statement should provide the following information, where applicable:

- Accession codes, unique identifiers, or web links for publicly available datasets
- A list of figures that have associated raw data
- A description of any restrictions on data availability

The datasets generated during the study are available from the corresponding authors upon request. Diffraction data were deposited at the Integrated Resource for Reproducibility in Macromolecular Crystallography (proteindiffraction.org) as entry SETD2_HR606-6vdb (https://proteindiffraction.org/project/SETD2_HR606_6vdb/). The current atomic model has been deposited in the Protein Data Bank (https://www.rcsb.org/) as entry 6VDB (https://www.rcsb.org/structure/6VDB). All biochemical data generated or analyzed during this study are included in this published article (and its supplementary information files). Source data are provided with this paper.

Field-specific reporting

Please select the one below that is the best fit for your research. If you are not sure, read the appropriate sections before making your selection.

☒ Life sciences ☐ Behavioural & social sciences ☐ Ecological, evolutionary & environmental sciences

For a reference copy of the document with all sections, see nature.com/documents/nr-reporting-summary-flat.pdf

Life sciences study design

All studies must disclose on these points even when the disclosure is negative.

Sample size	Experiments were done in duplicates or triplicates as indicated.
Data exclusions	N/A
Replication	Data are presented as mean ± SD. The number of experiments is indicated.
Randomization	N/A
Blinding	N/A

Reporting for specific materials, systems and methods

We require information from authors about some types of materials, experimental systems and methods used in many studies. Here, indicate whether each material, system or method listed is relevant to your study. If you are not sure if a list item applies to your research, read the appropriate section before selecting a response.

Materials & experimental systems

n/a	Involved in the study	
☐	☒	Antibodies
☐	☒	Eukaryotic cell lines
☒	☐	Palaeontology
☒	☐	Animals and other organisms
☒	☐	Human research participants
☒	☐	Clinical data

Methods

n/a	Involved in the study	
☒	☐	ChIP-seq
☐	☒	Flow cytometry
☒	☐	MRI-based neuroimaging

Antibodies

Antibodies used	For western blot: H3K36me3 (ab9050; lot. GR3257952-1) or GFP antibody (Clontech; lot. 1404005), anti-rabbit HRP (GE Healthcare; lot. 9739638).
Validation	Antibodies were validated by the manufacturers. Specificity of the H3K36me3 Ab was validated by in house Celluspot analysis and Western blot assays with controls.

Eukaryotic cell lines

Policy information about cell lines

Cell line source(s)	HEK293 cells were obtained from DSMZ, 38124 Braunschweig, GERMANY
Authentication	This was done by the provider.
Mycoplasma contamination	Every 1-2 month, a routine examination of cell lines in culture for any possible mycoplasma contamination was carried out using MycoAlert Mycoplasma Detection Kit (Lonza).
Commonly misidentified lines (See ICLAC register)	N/A

Flow Cytometry

Plots

Confirm that:

☒ The axis labels state the marker and fluorochrome used (e.g. CD4-FITC).

☒ The axis scales are clearly visible. Include numbers along axes only for bottom left plot of group (a 'group' is an analysis of identical markers).

☒ All plots are contour plots with outliers or pseudocolor plots.

☒ A numerical value for number of cells or percentage (with statistics) is provided.

Methodology

Sample preparation	N/A
Instrument	MACSQuant VYB, Miltenyi Biotec
Software	FlowJo software (Treestar)
Cell population abundance	N/A
Gating strategy	Standard gating was used for dsRed.

☒ Tick this box to confirm that a figure exemplifying the gating strategy is provided in the Supplementary Information.
